# Deep learning-based radiomics model from pretreatment ADC to predict biochemical recurrence in advanced prostate cancer

**DOI:** 10.3389/fonc.2024.1342104

**Published:** 2024-02-27

**Authors:** Huihui Wang, Kexin Wang, Yaofeng Zhang, Yuke Chen, Xiaodong Zhang, Xiaoying Wang

**Affiliations:** ^1^Department of Radiology, Peking University First Hospital, Beijing, China; ^2^School of Basic Medical Sciences, Capital Medical University, Beijing, China; ^3^Beijing Smart Tree Medical Technology Co. Ltd., Beijing, China; ^4^Department of Urology, Peking University First Hospital, Beijing, China

**Keywords:** prostate cancer, biochemical recurrence, apparent diffusion coefficient, radiomics, deep learning

## Abstract

**Purpose:**

To develop deep-learning radiomics model for predicting biochemical recurrence (BCR) of advanced prostate cancer (PCa) based on pretreatment apparent diffusion coefficient (ADC) maps.

**Methods:**

Data were collected retrospectively from 131 patients diagnosed with advanced PCa, randomly divided into training (n = 93) and test (n = 38) datasets. Pre-treatment ADC images were segmented using a pre-trained artificial intelligence (AI) model to identify suspicious PCa areas. Three models were constructed, including a clinical model, a conventional radiomics model and a deep-radiomics model. The receiver operating characteristic (ROC), precision-recall (PR) curve and decision curve analysis (DCA) were used to assess predictive performance in test dataset. The net reclassification index (NRI) and integrated discrimination improvement (IDI) were employed to compare the performance enhancement of the deep-radiomics model in relation to the other two models.

**Results:**

The deep-radiomics model exhibited a significantly higher area under the curve (AUC) of ROC than the other two (*P* = 0.033, 0.026), as well as PR curve (AUC difference 0.420, 0.432). The DCA curve demonstrated superior performance for the deep-radiomics model across all risk thresholds than the other two. Taking the clinical model as reference, the NRI and IDI was 0.508 and 0.679 for the deep-radiomics model with significant difference. Compared with the conventional radiomics model, the NRI and IDI was 0.149 and 0.164 for the deep-radiomics model without significant difference.

**Conclusion:**

The deep-radiomics model exhibits promising potential in predicting BCR in advanced PCa, compared to both the clinical model and the conventional radiomics model.

## 1 Introduction

Prostate cancer (PCa) is the second most common cancer in men and cancer-related mortality ranks the fifth (1). Treatment options include active surveillance, radical prostatectomy (RP), radiotherapy (RT), hormonal therapy (HT), chemotherapy, immunotherapy and others (2). After treatment, some patients might experience biochemical recurrence (BCR) (3). BCR serves as a prognostic indicator for the cure of PCa, clinical metastases and ultimately PCa-related death (3). Prediction of BCR could help healthcare professionals in treatment planning by identifying patients who may benefit from additional therapies or interventions.

Various prediction models for BCR incorporate clinical variables such as age, prostate specific antigen (PSA) level, clinical stage, Gleason score, and other relevant factors (3–8). These clinical variables are believed to be associated with the aggressiveness of PCa, which explains their usefulness in predicting BCR. Magnetic resonance(MR) image features have also been found to be associated with the aggressiveness of PCa (9). Therefore, it is reasonable to expect that utilizing MR image features can contribute to the prediction of BCR (10). In studies focusing on MR prediction models, the use of radiomics methods enables the extraction of more information compared to human image interpretation alone (11, 12). Radiomics allows for the analysis of intricate quantitative image features that may not be readily apparent to the human eyes, thereby enhancing the predictive capabilities (13–18).

However, previous research has primarily focused on localized PCa treated with RP and/or RT. Patients with advanced PCa who received only HT or complex treatment were not included in analysis, especially those with lymph node metastasis or distant metastasis. Additionally, previous MR radiomics studies predominantly relied on manual annotations of regions of interest (ROI) by experts to construct the prediction models. This manual annotation approach is time-consuming and labor-intensive and hampers the widespread clinical application of these models. Moreover, most previous MR radiomics research involved the extraction and analysis of imaging features using generic morphological, textural, and statistical features defined by predetermined formulas (19). While these features provide useful information, they may not fully capture the intricate patterns and relationships presented within the images. Recently, deep learning methods have shown promising applications in automating the feature extraction process and capturing more complex patterns and relationships within images (19–21). Deep learning models of PCa were mainly applied in preclinical discovery (22), Gleason grading (23), tumor metastasis (24) and BCR in RP (25). Thus, our study was aimed to develop deep learning model with automatic segmentation derived from pretreatment apparent diffusion coefficient (ADC) maps that may be predictive of BCR in advanced PCa.

## 2 Methods

### 2.1 Data enrollment

This retrospective study received approval from the local institutional review board of the Peking University First Hospital Medical Science Research ethics committee (IRB number: 2021-342), and the requirement for written consent was waived.

A total of 2,232 patients suspected of PCa between 2016 and 2020 in our institution were included in the study. The inclusion criteria were as follows: (a) Availability of pretreatment MR images in the Picture Archiving and Communication Systems (PACS). (b) Clinically diagnosed as advanced stage and initial treatment with RT, HT, or a combination of both. (c) Regular follow-up at least every three months in the first year and every six months in the second year. (d) Availability of clinical data. (e) Follow-up period of at least two years with documented biochemical recurrence (BCR+) or non-BCR (BCR-). BCR was defined as any PSA increase greater than 2 ng/mL compared to the PSA nadir value for patients who underwent RT with or without HT. For patients who received only HT, BCR was defined as castrate serum testosterone less than 1.7 nmol/L with either three consecutive PSA increases at least one week apart, resulting in a two-fold increase exceeding the nadir by 50% and a PSA greater than 2 ng/mL, or radiological progression evidenced by the appearance of new lesions (26).

**Figure 1:**
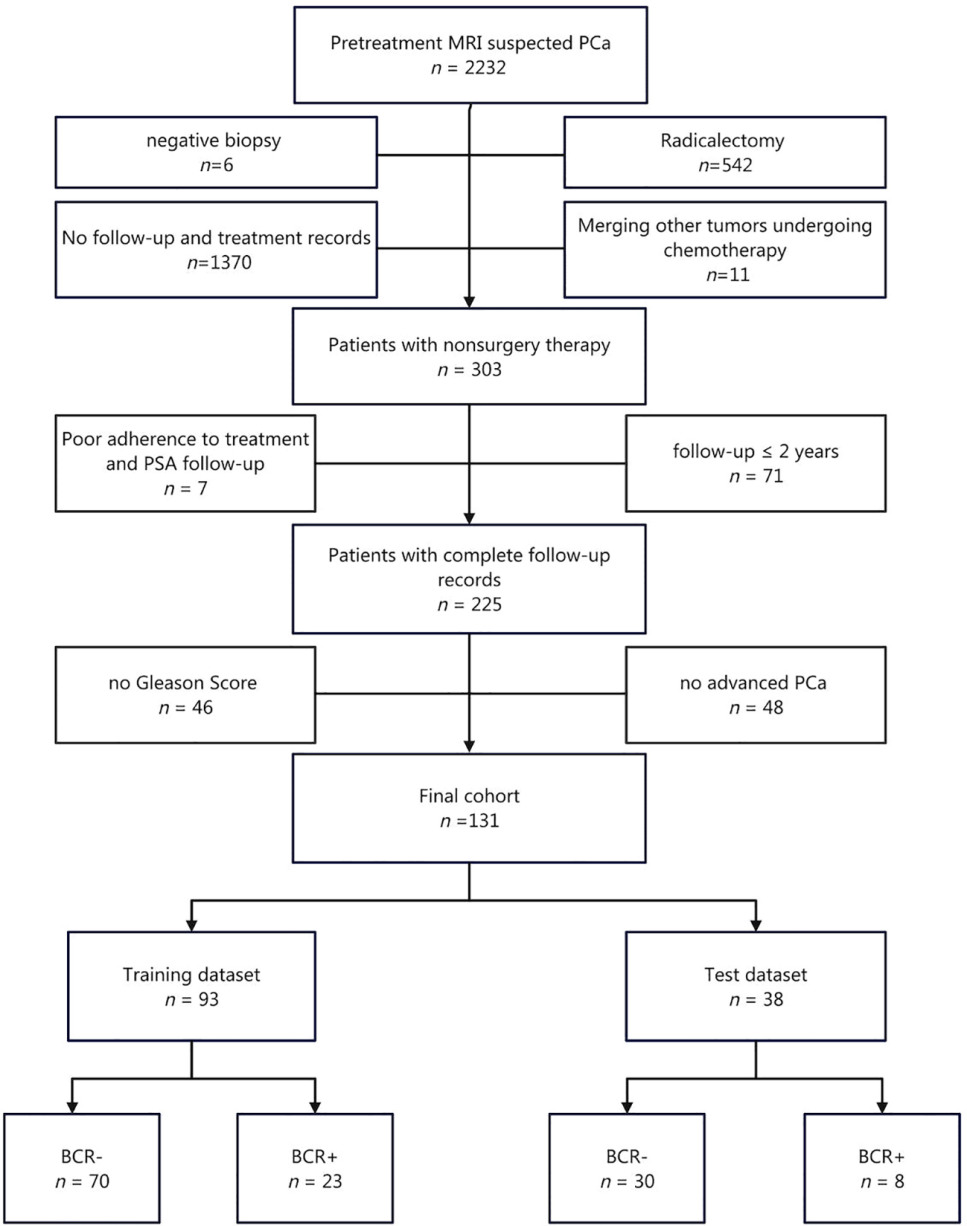
Flow chart of patient enrollment.

Finally, a total of 131 consecutive patients were included in this study, consisting of 100 BCR- and 31 BCR+ patients (**Figure 1**). Of these patients, 4 received only RT (including 4 BCR- and 0 BCR+), 75 received RT with HT (74 BCR- and 1 BCR+), and 52 received only HT (22 BCR- and 30 BCR+).

### 2.2 MRI acquisition parameters

A comprehensive description of acquisition parameters for seven different MR scanners are provided in **Table 1**. The magnetic field strength of MR scanners included 1.5 T (n = 46, 35.1%) and 3.0 T (n = 85, 64.9%). The radiomics analysis exclusively focused on ADC images, which were obtained with a median *b* value of 1400 s/mm^2^. Diffusion weighted imaging (DWI) was acquired by using single-shot echo planner imaging (SS-EPI) sequence. The calculation of ADC maps was performed using vendor-specific software associated with each MR scanner (GE: Advantage Workstation, Philips: IntelliSpace Portal, Siemens: syngo.via, UIH: uWS-MR Advanced Postprocess Workstation). Other imaging sequences, including T1-weighted imaging (T1WI), T2-weighted imaging (T2WI) and dynamic contrast enhancement (DCE), were acquired simultaneously but were not subjected to analysis in current study.

**Table 1:** Image acquisition protocols of ADC maps at seven MR scanners.

	GE	Philips	Siemens	UIH	Overall
	(N=77)	(N=22)	(N=30)	(N=2)	(N=131)
Magnetic field					
1.5 T	5 (6.5%)	11 (50.0%)	30 (100%)	0 (0%)	46 (35.1%)
3.0 T	72 (93.5%)	11 (50.0%)	0 (0%)	2 (100%)	85 (64.9%)
Model name					
Achieva	0 (0%)	9 (40.9%)	0 (0%)	0 (0%)	9 (6.9%)
Aera	0 (0%)	0 (0%)	30 (100%)	0 (0%)	30 (22.9%)
Discovery MR750	69 (89.7%)	0 (0%)	0 (0%)	0 (0%)	69 (52.7%)
Ingenia	0 (0%)	2 (9.1%)	0 (0%)	0 (0%)	2 (1.5%)
Multiva	0 (0%)	11 (50.0%)	0 (0%)	0 (0%)	11 (8.4%)
Signa Excite	8 (10.3%)	0 (0%)	0 (0%)	0 (0%)	8 (6.1%)
uMR 790	0 (0%)	0 (0%)	0 (0%)	2 (100%)	2 (1.5%)
*b* value (s/mm^2^)					
Median [Q1, Q3]	1400 [1400,1400]	1400 [1000,1400]	1400 [1400,1400]	1400 [1400,1400]	1400 [1400,1400]
Repetition time (ms)					
Median [Q1, Q3]	2660 [2640,3000]	2510 [2000,3410]	5010 [5010,5010]	3000 [2400,3000]	3000 [2640,5010]
Echo time (ms)					
Median [Q1, Q3]	61.3 [60.8,62.0]	71.0 [66.9,74.9]	53.0 [53.0,53.0]	67.0 [60.0,67.0]	61.2 [58.5,63.3]
Pixel bandwidth (MHz)					
Median [Q1, Q3]	1950 [1950,1950]	2010 [1790,2590]	1090 [1090,1090]	1390 [1390,1790]	1950 [1390,1950]
Slice thickness (mm)					
Median [Q1, Q3]	4.00 [4.00,4.50]	4.00 [4.00,5.00]	4.00 [4.00,4.00]	3.00 [3.00,3.00]	4.00 [4.00,4.50]
Slice spacing (mm)					
Median [Q1, Q3]	4.00 [4.00,4.50]	4.00 [4.00,5.00]	4.00 [4.00,4.00]	3.30 [3.15,3.30]	4.00 [4.00,4.50]
Reconstruction diameter (mm)					
Median [Q1, Q3]	240 [240,240]	240 [224,268]	200 [200,200]	200 [200,210]	240 [200,240]
Pixel spacing (mm)					
Median [Q1, Q3]	0.938 [0.938,0.938]	1.02 [0.961,1.15]	2.08 [2.08,2.08]	1.04 [0.736,1.04]	0.938 [0.938,1.38]

### 2.3 Region of interest

**Figure 2:**
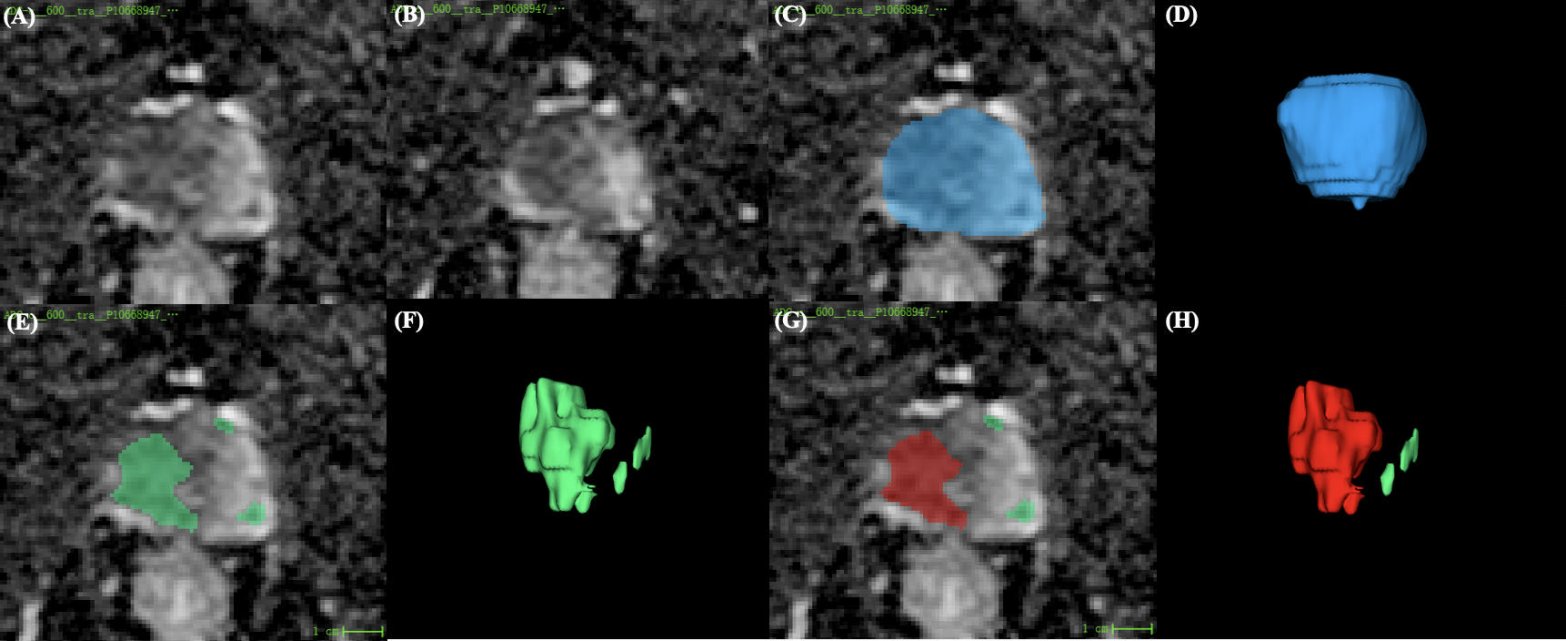
Illustration of an ROI example. **(A, B)** Two different slices of the ADC map. **(C,D)** AI model’s prediction of the prostate region (blue zone). **(E,F)** AI model’s detection of suspicious lesion areas (green zone). **(G,H)** The largest lesion (red zone) was selected for radiomics modeling. ROI, region of interest; ADC, apparent diffusion coefficient.

The areas of PCa were predicted by priorly trained models (22) on the ADC maps (**Figure 2**). Initially, the areas corresponding to prostate gland were segmented by artificial intelligence (AI) models. Subsequently, the regions of suspected PCa were segmented in a sequential manner. The three-dimensional volume ROI was utilized for extracting image features required for model development. If multiple PCa foci were segmented, the largest one was automatically taken as the ROI. Prostate Imaging and Reporting Archiving Data System (PI-RADS) v2.1 was used to score lesions of interest by an experienced radiologist (work experience more than 10 years). Among 131 patients, 106 had only one suspicious lesion, 20 had two, 4 had three, and 1 had four. The volume, location, and PI-RADS scores of these lesions are detailed in **Table 2**. In 121 patients, the largest lesion coincided with the one rated as the highest PI-RADS score. In 10 cases, the largest lesion shared the same PI-RADS score as the second largest lesion.

**Table 2:** Lesions detected by the AI model.

	Patient with 1 lesion	Patient with 2 lesions	Patient with 3 lesions	Patient with 4 lesions
**Total patients (n)**	106	20	4	1
**Total lesions (n)**	106	40	12	4
PCa location				
PZ and TZ	96 (90.6%)	15 (37.5%)	3 (25.0%)	1 (25.0%)
PZ only	8 (7.5%)	15 (37.5%)	8 (66.7%)	1 (25.0%)
TZ only	2 (1.9%)	10 (25.0%)	1 (8.3%)	2 (50.0%)
PCa volume (mm^3^)				
Median [Min, Max]	15300 [288, 102000]	756 [52, 44324]	632 [107, 4581]	286 [103, 1793]
PI-RADS score				
3	0 (0%)	3 (7.5%)	0 (0%)	1 (25.0%)
4	8 (7.5%)	19 (47.5%)	9 (75.0%)	1 (25.0%)
5	98 (92.5%)	18 (45.0%)	3 (25.0%)	2 (50.0%)
Largest lesion and highest PI-RADS score consistency				
Consistency	106 (100%)	12 (60.0%)	3 (75.0%)	0 (0%)
Inconsistency	0 (0%)	8 (40.0%)	1 (25.0%)	1 (100%)

### 2.4 Model development

The collected data were divided into two datasets, namely, the training dataset (n = 93) and the test dataset (n = 38), using a random allocation with a ratio of 7:3 (27). Subsequently, three prediction models were developed using the training dataset: a clinical model, a conventional radiomics model, and a deep-radiomics model. The clinical characteristics, including age, PSA level, PI-RADS score, International Society of Urological Pathology (ISUP) group for biopsy pathology, clinical stage and treatment, remained consistent across all models. However, the image features varied among models, and their specific extraction methods will be elaborated in the subsequent manuscript.

To construct the clinical model, location, volume and ADC value of PCa area segmented by the AI models were selected as image features. Univariable logistic regression and multivariable logistic regression analysis were conducted to identify the significant predictors. The selection of predictors was performed using a stepwise selection approach guided by the Akaike information criterion (AIC). The adequacy of the model was evaluated by calculating the R2 value using the Nagelkerke method. To help comprehensive interpretation of the model, a nomogram graph was generated.

To construct the conventional radiomics model, the image features were extracted from ROIs on the ADC maps using the Pyradiomics package in Python (https://pyradiomics.readthedocs.io/en/latest/changes.html). To account for potential variations across different scanners, preprocessing of the ADC maps was performed by applying image normalization to mitigate confounding effects. Three types of images were analyzed: “Original Images” representing unfiltered images, “LoG Images” obtained by applying the Laplacian of Gaussian filter, and “Wavelet Images” generated through a three-dimensional wavelet transformation using the PyWavelet package in the x, y, and z directions. The ROIs were then preprocessed to ensure a consistent size. A total of 14 shape features, 18 first-order statistical features, and 24 texture features were extracted from the images. The shape features were exclusively extracted from the “Original Images,” while the first-order statistical and texture features were extracted from all three types of images. Therefore, a total of 14 shape features, 216 first-order statistical features, and 840 texture features were obtained. The mathematical expressions and semantic meanings of the features extracted can be found at https://pyradiomics.readthedocs.io/en/latest/. After feature extraction, several additional steps were performed. Z score normalization was applied to rescale the extracted features, and Pearson correlation coefficients (PCCs) were calculated to identify highly correlated features. Features with a PCC value exceeding 0.99 were removed to mitigate multicollinearity. Analysis of variance (ANOVA) was then used to select features for the final radiomics model. As the classifier, eXtreme gradient boosting (XGBoost) algorithm was employed. And SHAP (SHapley Additive exPlanations) values were used to interpret the results obtained from the XGBoost models.

To construct the deep-radiomics model, the image features were extracted by employing a deep learning algorithm. The construction process involved several steps. First, the intensities of ADC maps were preprocessed by normalizing them. Second, ROIs were resampled to ensure a consistent voxel size. Third, a pre-trained deep learning model was utilized to extract features from ROIs, leveraging the power of learned representations. Specifically, the ROIs were input into the convolutional layers of the MedicalNet architecture, which had been initialized with pre-trained weights to extract discriminative features. A comprehensive description of the network can be found in previous work (https://github.com/Tencent/MedicalNet). The resulting channel feature maps were then subjected to feature dimension reduction by filtering with the maximum value. This process yielded a set of 2048 one-dimensional features. After extracting deep features, the construction of the deep-radiomics model followed a similar procedure to the conventional radiomics model.

The parameters used for feature extraction, feature selection, internal validation, and model building are presented in the **Supplementary Material**.

### 2.5 Model evaluation

Multiple metrics were employed to evaluate of the constructed models performance using the test dataset. First, the receiver operating curve (ROC) analysis with the use of the area under the curve (AUC) was utilized as a widely accepted metric.

Because of the imbalanced distribution of the BCR- and BCR+ cases, the precision-recall (PR) curve was employed. The AUC of the PR and 95% bootstrap confidence interval (BCa) were calculated. If the BCa does not include zero, it indicates that the difference in PR AUC between the models is statistically significant.

To gain insights into the clinical utility of the models, decision curve analysis (DCA) was employed. The net reclassification index (NRI) and integrated discrimination improvement (IDI) were utilized to quantify the enhancement in prediction accuracy achieved by the radiomics and deep-radiomics models compared to the clinical model. A positive NRI indicates improved risk classification, while a negative NRI suggests misclassification. A higher IDI value indicates enhanced discrimination between individuals with and without the event of interest.

### 2.6 Statistical analysis

IBM SPSS® 20.0 (www.ibm.com), MedCalc 20.014 (www.medcalc.org) and R 3.5.1 (www.r-project.org) were used for statistical analysis.

Descriptive statistics were used to summarize the data, with mean (standard deviation) reported of continuous variables that followed a normal distribution and median [Q1, Q3] of continuous variables that did not follow a normal distribution. Categorical variables were reported as frequencies (percentage%). A Mann-Whitney U test or chi-square test was used to assess differences in clinical characteristics both between the training and test cohorts and between the BCR- and BCR+ groups. The DeLong test was used to compare the difference between the AUCs of ROC. The level of statistical significance was set at *P* < 0.05.

## 3 Results

### 3.1 Clinical characteristics

Out of 131 cases, 62 (47.3%) were classified as locally advanced PCa, while 20 (15.3%) exhibited lymph node metastasis, and 49 (37.4%) presented with distant metastasis. There was no significant difference in any feature between the training and test datasets (**Table 3**). BCR rates were 24.7% (23/93) in the training cohort and 21.1% (8/38) in the test cohort, without significant difference (*P* = 0.824).

**Table 3:** Clinical characteristics of the training and test datasets.

	Overall	Training dataset	Test dataset	*P* value
	(N=131)	(N=93)	(N=38)	
Age (year)				
Mean (SD)	71.9 (7.66)	71.3 (8.09)	73.4 (6.34)	0.152
tPSA (ng/dL)				
Median [Q1, Q3]	42.6 [14.2,124]	43.5 [14.3,124]	27.3 [13.8,94.0]	0.505
PI-RADS score				
4	14 (10.7%)	10 (10.8%)	4 (10.5%)	>0.999
5	117 (89.3%)	83 (89.2%)	34 (89.5%)	
PCa location				
PZ and TZ	115 (87.8%)	84 (90.3%)	31 (81.6%)	0.297
PZ only	10 (7.6%)	5 (5.4%)	5 (13.2%)	
TZ only	6 (4.6%)	4 (4.3%)	2 (5.3%)	
PCa volume (mm^3^)				
Median [Q1, Q3]	10000 [2750,25100]	12000 [2880,26400]	6140 [2200,19400]	0.373
PCa ADC value (*10^-3 s/mm^3^)				
Mean (SD)	791 (128)	790 (124)	793 (138)	0.821
ISUP				
1	4 (3.1%)	4 (4.3%)	0 (0%)	0.557
2	12 (9.2%)	7 (7.5%)	5 (13.2%)	
3	20 (15.3%)	15 (16.1%)	5 (13.2%)	
4	27 (20.6%)	18 (19.4%)	9 (23.7%)	
5	68 (51.9%)	49 (52.7%)	19 (50.0%)	
Stage				
T3~4N0M0	62 (47.3%)	43 (46.2%)	19 (50.0%)	0.316
TxN1M0	20 (15.3%)	17 (18.3%)	3 (7.9%)	
TxNxM1	49 (37.4%)	33 (35.5%)	16 (42.1%)	
Treatment				
Radiotherapy	4 (3.1%)	1 (1.1%)	3 (7.9%)	0.104
Hormonal therapy	52 (39.7%)	39 (41.9%)	13 (34.2%)	
Comprehensive therapy	75 (57.3%)	53 (57.0%)	22 (57.9%)	
Label				
BCR-	100 (76.3%)	70 (75.3%)	30 (78.9%)	0.824
BCR+	31 (23.7%)	23 (24.7%)	8 (21.1%)	

Compared the BCR+ and BCR- groups (**Table 4**), no significant difference was found in age, PI-RADS score, PCa location, PCa ADC value, and ISUP group (all *P* > 0.05). There were significant differences in PSA level, PCa volume, clinical stage and treatment method between the BCR- and BCR+ groups (all *P* < 0.05).

**Table 4:** Clinical characteristics of the BCR- and BCR+ datasets.

	BCR-	BCR+	*P* value
	(N=100)	(N=31)	
Age (year)			
Mean (SD)	72.3 (7.55)	70.6 (8.00)	0.335
tPSA (ng/dL)			
Median [Q1, Q3]	36.8 [13.9,95.9]	75.2 [17.8,472]	0.044
PI-RADS score			
4	12 (12.0%)	2 (6.5%)	0.589
5	88 (88.0%)	29 (93.5%)	
PCa location			
PZ and TZ	8 (8.0%)	2 (6.5%)	0.352
PZ only	6 (6.0%)	0 (0%)	
TZ only	86 (86.0%)	29 (93.5%)	
PCa volume (mm^3^)			
Median [Q1, Q3]	6190 [2240,19500]	23800 [5760,41300]	0.004
PCa ADC value (*10^-3 s/mm^3^)			
Mean (SD)	802 (133)	756 (103)	0.111
ISUP			
1	2 (2.0%)	2 (6.5%)	0.077
2	12 (12.0%)	0 (0%)	
3	17 (17.0%)	3 (9.7%)	
4	22 (22.0%)	5 (16.1%)	
5	47 (47.0%)	21 (67.7%)	
Stage			
TxN0M0	55 (55.0%)	7 (22.6%)	<0.001
TxN1M0	18 (18.0%)	2 (6.5%)	
TxNxM1	27 (27.0%)	22 (71.0%)	
Treatment			
Radiotherapy	4 (4.0%)	0 (0%)	<0.001
Hormonal therapy	22 (22.0%)	30 (96.8%)	
Comprehensive therapy	74 (74.0%)	1 (3.2%)	

### 3.2 Model development

**Figure 3:**
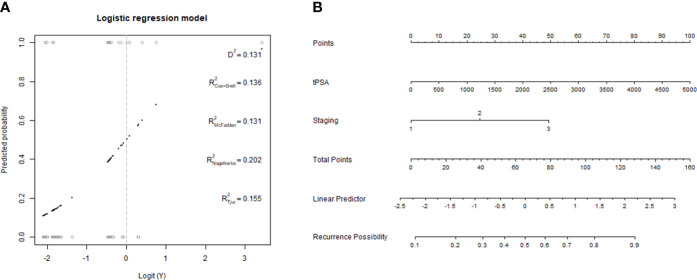
Development of clinical model. **(A, B)** a generalized linear model and the nomogram of the clinical model.

**Figure 4:**
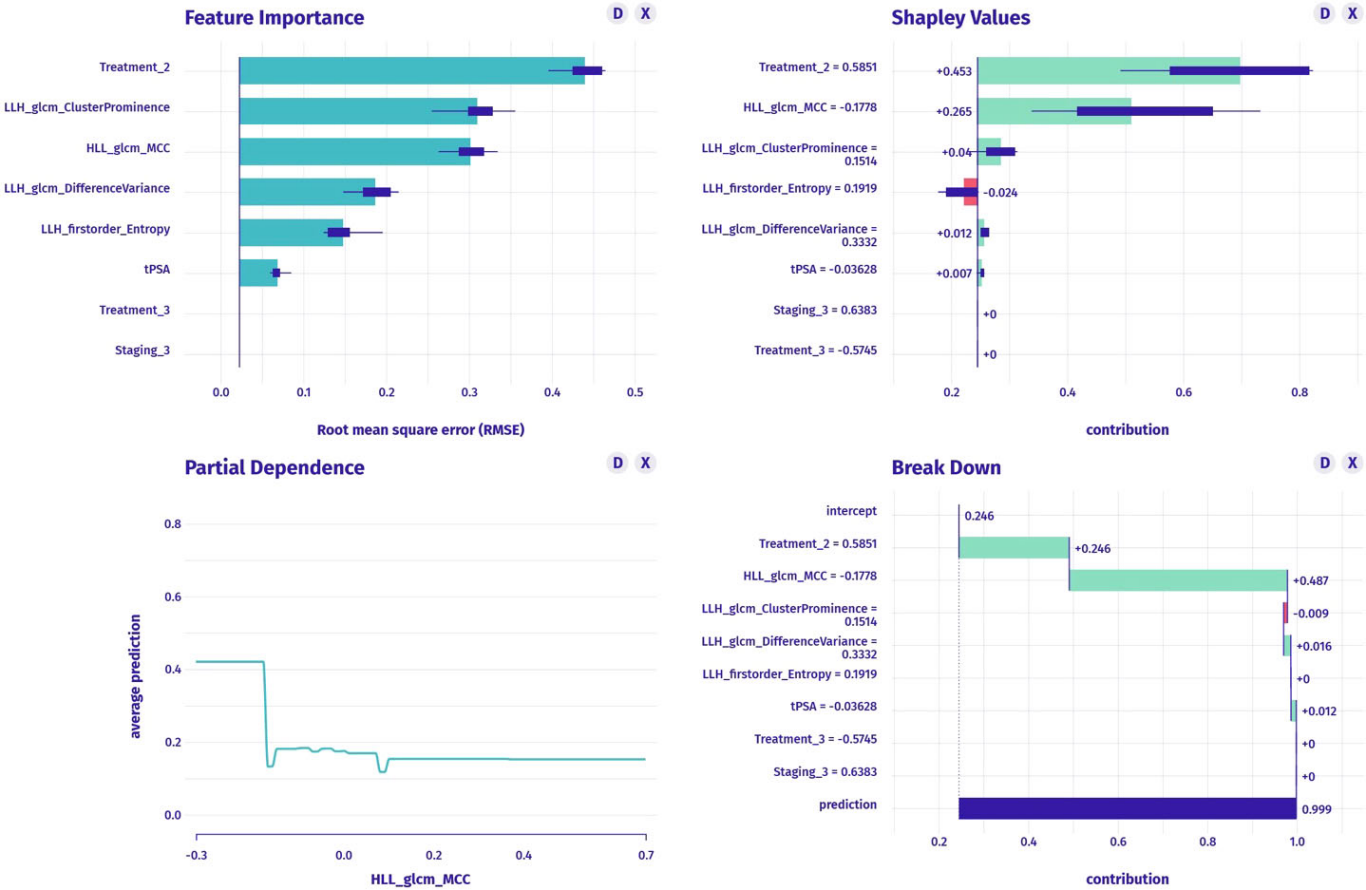
The construction of conventional radiomics model, including feature importance (upper left graph), SHAP values (upper right graph), partial dependence plot (lower left graph) and breakdown plot (lower right graph).

**Figure 5:**
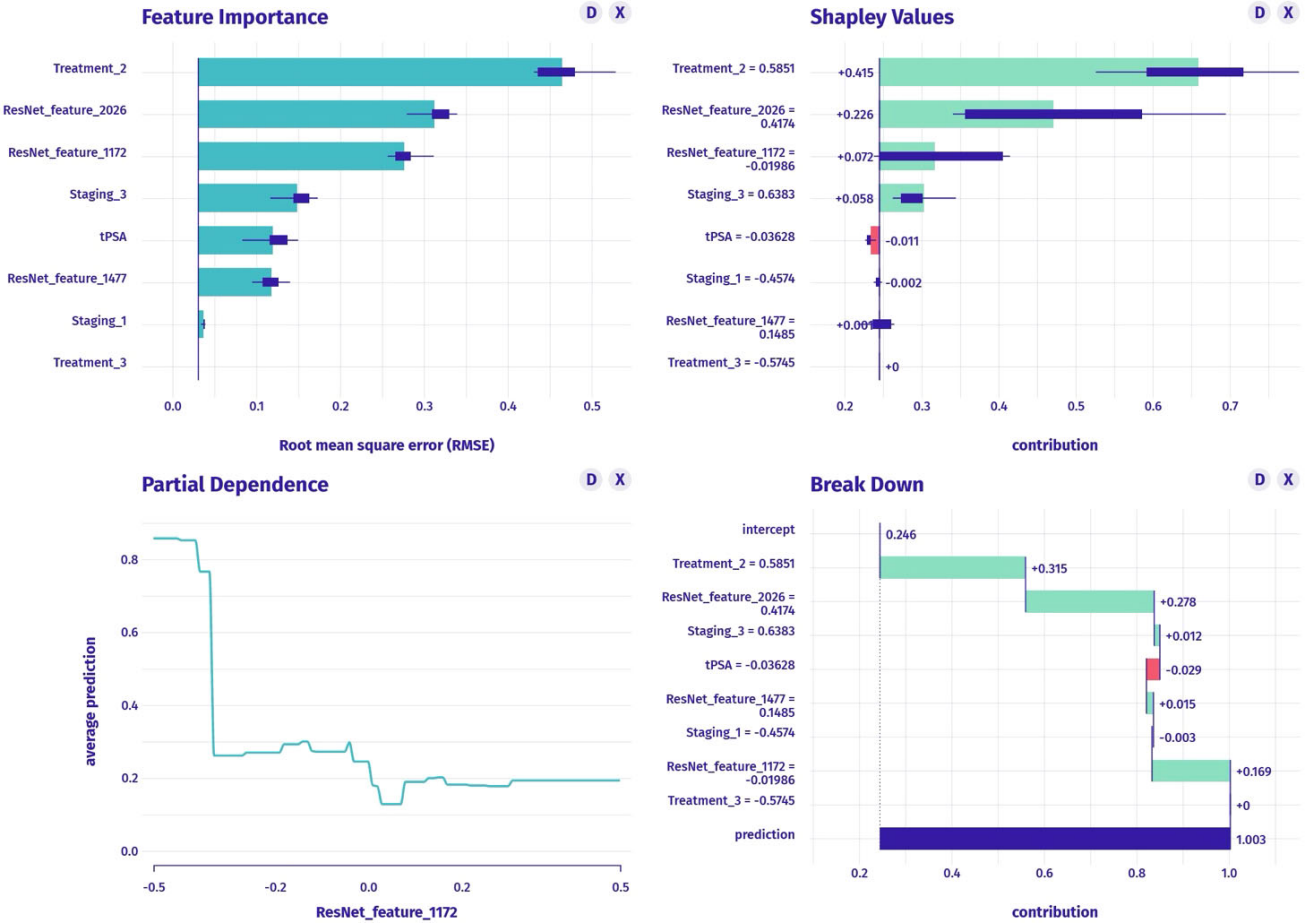
The construction of deep-radiomics model, including feature importance (upper left graph), SHAP values (upper right graph), partial dependence plot (lower left graph) and breakdown plot (lower right graph).

The clinical model was constructed using stepwise multivariable logistic regression. PSA and stage were the final predictor variables included in the model after selection process (**Table 5**, **Figure 3**). The conventional radiomics model incorporated PSA, stage, treatment, and four image features extracted from ADC maps as the final predictor variables (**Figure 4**). Similarly, the deep-radiomics model included PSA, stage, treatment, and three image features extracted using a deep learning algorithm as the final predictor variables (**Figure 5**).

**Table 5:** Odds ratios of the logistic regression models.

Parameter	Description	Odds ratio (univariable, 95% CI)	Odds ratio (multivariable, 95% CI)
**Age**	Mean ± SD	0.97 (0.91-1.03, *P* = 0.313)	
**tPSA**	Mean ± SD	1.00 (1.00-1.00, *P* = 0.059)	1.00 (1.00-1.00, *P* = 0.276)
**PI-RADS score**	4	Reference	
	5	1.35 (0.27-6.89, *P* = 0.714)	
**ISUP**	1	Reference	
	2	0.00 (0.00-Inf, *P* = 0.991)	
	3	0.25 (0.02-2.58, *P* = 0.244)	
	4	0.20 (0.02-2.03, *P* = 0.174)	
	5	0.44 (0.06-3.43, *P* = 0.434)	
**Stage**	TxN0M0	Reference	Reference
	TxN1M0	0.82 (0.15-4.54, *P* = 0.822)	0.79 (0.14-4.36, *P* = 0.783)
	TxNxM1	5.14 (1.71-15.46, *P* = 0.004)	4.02 (1.25-12.90, *P* = 0.019)
**Treatment**	Radiotherapy	Reference	
	Hormonal therapy	7.45*10^6 (0.00-Inf, *P* = 0.991)	
	Comprehensive therapy	1.11*10^5 (0.00-Inf, *P* = 0.994)	
**PCa ADC value**	Mean ± SD	1.00 (0.99-1.00, *P* = 0.437)	
**PCa volume**	Mean ± SD	1.00 (1.00-1.00, *P* = 0.061)	

### 3.3 Model evaluation

**Figure 6:**
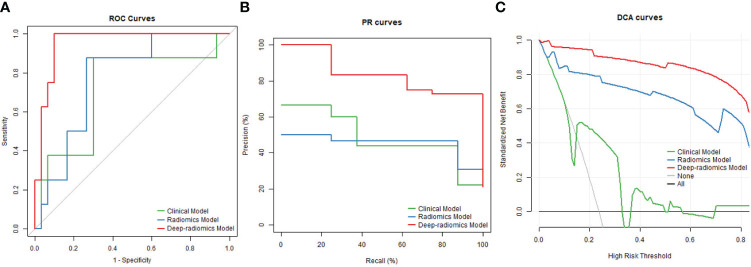
ROC **(A)**, RP **(B)** and DCA **(C)** curves for three predictive models in the test dataset.

The AUC of the ROC was 0.717 (95% CI: 0.492, 0.941) for the clinical model, 0.771 (95% CI: 0.607, 0.935) for the conventional radiomics model, and 0.954 (95% CI: 0.892, 1.000) for the deep-radiomics model in the test dataset (**Figure 6A**). The deep-radiomics model exhibited a significantly higher AUC than the clinical model (*P* = 0.033) and the conventional radiomics model (*P* = 0.026). However, there was no significant difference between the AUC of the clinical model and the conventional radiomics model (*P* = 0.570).

The AUC of the PR curve was 0.385 (95% CI: 0.191, 0.696) for the clinical model, 0.373 (95% CI: 0.209, 0.665) for the conventional radiomics model, and 0.805 (95% CI: 0.451, 0.985) for the deep-radiomics model (**Figure 6B**). The PR AUC difference between the clinical model and the deep-radiomics model was 0.420 (95% BCa: 0.292, 0.651), indicating a statistically significant distinction. Similarly, the PR AUC difference between the conventional radiomics model and the deep-radiomics model was 0.432 (95% BCa: 0.327, 0.638), demonstrating statistical significance. However, the PR AUC difference between the clinical model and the conventional radiomics model was 0.011 (95% BCa: -0.070, 0.216), revealing no statistical significance.

The DCA curve demonstrated superior performance of the deep-radiomics model compared to the conventional radiomics model and the clinical model across all risk thresholds. Additionally, the DCA curve of the conventional radiomics model outperformed the clinical model across all risk thresholds (**Figure 6C**).

Compared to the clinical model, the conventional radiomics model demonstrated an NRI of 0.359 (95% CI: 0.099, 0.973), and the deep-radiomics model showed a higher NRI of 0.508 (95% CI: 0.309, 1.175). Compared to the conventional radiomics model, the deep-radiomics model showed an NRI of 0.149 (95% CI: 0.032, 0.300).

When using the clinical model as reference, the conventional radiomics model exhibited an IDI of 0.516 (95% CI: 0.294, 0.666), and the deep-radiomics model showed a higher IDI of 0.679 (95% CI: 0.431, 0.795). When comparing the conventional radiomics model to the deep-radiomics model, the IDI was 0.164 (95% CI: -0.045, 0.363) without statistical difference.

## 4 Discussion

In the current study, we introduced a clinical model and two radiomics models demonstrating favorable accuracy in predicting BCR for patients with advanced PCa and the deep-radiomics model showed the most excellent performance. Several clinical prediction models have been proposed (3–8), and MR radiomics models also showed promising results (10, 13–18). Our findings align with previous studies in terms of the predictive efficiency of radiomics models for BCR. However, our study differs from previous research in three main aspects: (1) patients with advanced PCa who received non-surgical treatment as research objective, (2) the utilization of a pre-trained AI model for automatic segmentation of the ROI, and (3) a comparison between conventional radiomics models and deep learning radiomics models in terms of feature extraction.

MR radiomics models have demonstrated diverse applications in the detection, classification and management of PCa (28, 29). However, studies specifically focusing on radiomics models for predicting BCR remain limitation and exist inconsistency in the methodologies (13–18). A review of literature indicates that a variety of MR imaging sequences were used in BCR prediction models, such as MR perfusion (13), T2WI (15, 17), ADC maps (14), a combination of T2WI and ADC maps (14, 16) and combinations of T1WI with T2WI and DWI (14, 16). In these studies, different types of ROI were annotated, encompassing the prostate gland (13, 17, 18), prostate with an expanded margin (15) and prostate tumor (14–16). Notably, manual annotation methods were predominantly employed for ROI delineation (13, 16–18) and a small number of studies utilized semi-automatic annotation techniques (14, 15). These MR radiomics models demonstrated AUC values ranging from 0.63 to 0.85 for the prediction of BCR (13–18). Our study demonstrated that the three models had predictive performance (AUCs ranging from 0.717 to 0.954) comparable to or slightly better than previous studies. Additionally, a notable strength of this study was the utilization of an automatic annotation approach for ROIs using a pre-trained AI model. This automatic process addresses the inherent challenges associated with manual ROI annotation, such as the labor-intensive nature and the potential for intra- and inter-observer variability, which can compromise the robustness of radiomics models.

Another strength of our study is the comparison of image feature extraction methods in the radiomics pipeline. In the process of constructing radiomics models, the subsequent step after selecting ROI involves extracting image features for training the classifier (20). In our study, we developed two types of radiomics models: a conventional radiomics model and a deep-radiomics model. The key distinction between the two models lies in the approach of feature generation. The conventional radiomics model extracts standard features from ROIs calculated based on predefined formulas, whereas the deep-radiomics model employs deep network architectures to discover task-specific optimal features (21). Predictive features in the deep-radiomics model are learned independently during training, eliminating the need for explicit feature definitions. Given that the deep-radiomics model adapts to the data, it is reasonable to expect it to yield superior results compared to the conventional model. However, in 26% of previous studies, deep-radiomics models did not surpass conventional models (21). It reported that deep-radiomics models outperformed conventional radiomics models with a median increase in the AUC from 0.025 to 0.045. Since it is generally unknown which method will perform best in advance, it is recommended to test multiple methods as a best practice (30). It is inspiring that the predictive model that combined clinical characteristics, visual features, deep learning features, and radiomics features based on computed tomography (CT) or MR images showed improved predictive efficiency (31–33), while the purpose of our study was to find a simple and convenient way to predict the BCR in advanced PCa. If a large number of parameters and complex predictive methods were used, the complexity of operations may affect the efficiency of clinical work and limit predictive models promotion. Therefore, three separate predictive models were evaluated instead of combining them in this study.

In this study, we employed multiple methods to evaluate the performance of predictive models from different perspectives (34). The ROC analysis is commonly used in this type of research because it provides a measure of the overall discriminatory power of a model. However, in our study, PR evaluation was more informative than the ROC because of the imbalanced sample sizes of BCR+ and BCR-. It helps assess the model’s ability to correctly identify positive instances while minimizing false positives (35). While ROC and PR curve provide valuable insights into the model’s classification abilities, they do not directly consider real-world clinical scenarios or the specific context in which the model will be applied. DCA, on the other hand, takes into account the net benefit or harm associated with using a predictive model to guide clinical actions compared to other strategies or no action, thereby providing a more comprehensive evaluation of the model’s performance in terms of its clinical impact (35). Besides, NRI and IRI are commonly used in the field of predictive modeling and risk assessment (34). They provide a way to assess the added value of incorporating certain variables or features into a model compared to a baseline or reference model. They help evaluate to what extent the new model improves the classification or discrimination performance, thereby achieving better risk stratification or outcome prediction. Our results consistently showed that the deep-radiomics model outperformed the other two models in most aspects.

Our study has several inherent limitations that should be acknowledged. First, the retrospective nature conducted in a single center resulted in a relatively small cohort, reducing the statistical power and potentially limiting the generalizability of the findings. Indeed, this is a recurring problem in the field of radiomics research, as seen in examples of other sample sizes in the literature include 49 (36), 120 (15, 16) and 133 (17) patients. In an ideal manner, it is recommended to train an unbiased classifier with the same number of samples from both BCR+ and BCR- groups in the training dataset. The test dataset may be imbalanced, but it is not recommended to have an imbalanced training set. However, considering the distribution of our enrollment cases and previous studies (17, 18), it was not feasible in this study and may lead to inaccurate model parameters. Given AUC is a powerful indicator of classification performance in skewed datasets, we still obtain a reasonable AUC on the test dataset, indicating the robustness of radiomic features in predicting BCR. Our findings are encouraging, as this study provide preliminary evidence of the correlation between imaging and prognosis in advanced cancer patients. Besides, previous study has shown that the performance of the model may decrease due to heterogeneity in the collection protocols and patients with external data validation (37). So our results require a larger validation and external validation before these findings can be applied in the clinical practice. Further study would increase the patient sample size by extend inclusion time and potentially develop this study into a multicenter research project. Second, there was a possibility of selection bias in our study due to the influence of urologists’ and patient-related factors on treatment decisions, which were not fully captured in the data. The diverse treatment options and their impact on prognosis were not thoroughly analyzed. At last, the application of deep learning models in our study focused on a specific network architecture. The reproducibility and generalizability of deep networks remain uncertain, as they are known to be sensitive to initial weights and may exhibit erratic behavior. Further studies should explore the use of alternative network architectures to evaluate their performance.

## 5 Conclusion

Despite the above limitations, we can conclude that the deep-radiomics model, shows excellent accuracy in predicting BCR in advanced PCa, which may make an effect on treatment methods and subsequent therapeutic interventions. The deep-radiomics model was superior than the clinical model and the conventional radiomics model in the aspect of prediction accuracy, clinical impact and risk assessment.

## Data availability statement

The original contributions presented in the study are included in the article/**Supplementary Material**, further inquiries can be directed to the corresponding author/s.

## Ethics statement

The studies involving humans were approved by Ethics Committee of Peking University First Hospital. The studies were conducted in accordance with the local legislation and institutional requirements. The ethics committee/institutional review board waived the requirement of written informed consent for participation from the participants or the participants’ legal guardians/next of kin because this was a retrospective study.

## Author contributions

HW: Writing – review & editing, Data curation, Funding acquisition, Investigation, Validation, Visualization, Writing – original draft. KW: Writing – original draft, Formal analysis, Software. YZ: Software, Writing – original draft, Methodology, Validation. YC: Conceptualization, Investigation, Writing – review & editing. XZ: Writing – review & editing, Resources, Software, Validation. XW: Writing – review & editing, Conceptualization, Methodology, Project administration, Supervision.
