# Supplementary material for: Deep learning-based radiomics model from pretreatment ADC to predict biochemical recurrence in advanced prostate cancer
Source: Front Oncol. 2024 Feb 27;14:1342104. doi: 10.3389/fonc.2024.1342104 (PMC10928490; doi:10.3389/fonc.2024.1342104)
Supplement: Supplementary file 1 [file DataSheet_1.pdf]

Supplementary Materials

Part 1 Feature Extraction Parameters

1. Image normalization

MR image patterns may differ between scanners and vendors. By normalizing the image before feature calculation, this confounding effect may be reduced. The normalization scale was 100 with bin width 5, voxel array shift: 300.

To address potential variations arising from the use of different MRI scanners, we implemented a preprocessing protocol for the ADC maps. This protocol included image normalization, a crucial step in reducing confounding effects that might skew the analysis. Specifically, normalization was used to adjust the intensity values across the images, bringing them to a common scale and thus enhancing the comparability of images obtained from different scanners.

2. Image types

In our study, we analyzed three distinct types of images:

Original images: These are the unprocessed, raw images acquired directly from the MR scans. The data served as the baseline data for analysis.

"LoG images": These images are generated by applying the Laplacian of Gaussian (LoG) filter to the original images. The LoG filter, known for enhancing regions of rapid intensity change, is applied at three different scales to capture a range of textural details: fine (σ=1.0), medium (σ=3.0), and coarse (σ=5.0). Consequently, three distinct sets of LoG images are produced, each corresponding to one of these scales.

"Wavelet images": To obtain these images, the original images underwent a three-dimensional wavelet transformation in the x, y, and z directions using the PyWavelet package. Each transformation involves filtering the image with either a high or low bandpass filter in each direction. This process results in eight unique

combinations of high- and low-pass filtering, namely, LLH, LHL, HLL, LHH, HHL, HLH, HHH, and LLL, where 'H' denotes high-pass filtering and 'L' denotes low-pass filtering. Each combination represents a different wavelet-decomposed image, capturing various aspects of image texture and detail.

Finally, 3 types of images were used in this study: Original image (n = 1), LoG images (n = 3) and Wavelet images (n = 8).

3. Radiomics feature extraction

Following the generation of these image types, we standardized the ROI to ensure consistency in size across all images. We then proceeded with the extraction of a comprehensive set of features. There were 3 types of radiomics features used in this study: (1) shape-based features (n = 14); (2) first-order statistical features (n = 18); and (3) texture features (n = 24 Gray Level Co-occurrence Matrix (GLCM) +16 Gray Level Co-occurrence Matrix (GLRLM) +16 Gray Level Size Zone Matrix (GLSZM) +14 Gray Level Dependence Matrix (GLDM)).

The shape features were only extracted from the “Original Images”, while the first-order statistical and textural features were extracted from all three types of images (Table 1). Therefore, 14 shape features, 216 ((1 Original Images + 3 LoG Images + 8 Wavelet Images) × 18) first-order statistical features and 840 (1 Original Images + 3 LoG Images + 8 Wavelet Images) × (24+16+16+14)) texture features were used in our study. We extracted all or some of these features from the region of interest (ROI) as needed.

Table 1 Radiomic features used in this study

**Feature classification**	**Features names**
Shape Features (n=14)	
	Elongation

	Flatness
	Least Axis Length
	Major Axis Length
	Maximum 2D Diameter (Column)
	Maximum 2D Diameter (Row)
	Maximum 2D Diameter (Slice)
	Maximum 3D Diameter
	Mesh Volume
	Minor Axis Length
	Sphericity
	Surface Area
	Surface Volume Ratio
	Voxel Volume
First-order Statistical Features (n=18)	
	10th Percentile
	90th Percentile
	Energy
	Entropy
	Interquartile Range
	Kurtosis
	Maximum
	Mean Absolute Deviation
	Mean
	Median
	Minimum
	Range

	Robust Mean Absolute Deviation
	Root Mean Squared
	Skewness
	Total Energy
	Uniformity
	Variance
Texture Features	
GLCM (n=24)	
	Autocorrelation
	Cluster Prominence
	Cluster Shade
	Cluster Tendency
	Contrast
	Correlation
	Difference Average
	Difference Entropy
	Difference Variance
	Inverse Difference (ID)
	Inverse Difference Moment (IDM)
	Inverse Difference Moment Normalized (IDMN)
	Inverse Difference Normalized (IDN)
	Informational Measure of Correlation (IMC) 1
	Informational Measure of Correlation (IMC) 2
	Inverse Variance
	Joint Average
	Joint Energy
	Joint Entropy

	Maximal Correlation Coefficient (MCC)
	Maximum Probability
	Sum Average
	Sum Entropy
	Sum of Squares
GLRLM (n=16)	
	Gray Level Non-Uniformity (GLN)
	Gray Level Non-Uniformity Normalized (GLNN)
	Gray Level Variance (GLV)
	High Gray Level Run Emphasis (HGLRE)
	Long Run Emphasis (LRE)
	Long Run High Gray Level Emphasis (LRHGLE)
	Long Run Low Gray Level Emphasis (LRLGLE)
	Low Gray Level Run Emphasis (LGLRE)
	Run Entropy (RE)
	Run Length Non-Uniformity (RLN)
	Run Length Non-Uniformity Normalized (RLNN)
	Run Percentage (RP)
	Run Variance (RV)
	Short Run Emphasis (SRE)
	Short Run High Gray Level Emphasis (SRHGLE)
	Short Run Low Gray Level Emphasis (SRLGLE)
GLSZM (n=16)	
	Gray Level Non-Uniformity (GLN)
	Gray Level Non-Uniformity Normalized (GLNN)
	Gray Level Variance (GLV)
	High Gray Level Zone Emphasis (HGLZE)

	Large Area Emphasis (LAE)
	Large Area High Gray Level Emphasis (LAHGLE)
	Large Area Low Gray Level Emphasis (LALGLE)
	Low Gray Level Zone Emphasis (LGLZE)
	Size-Zone Non-Uniformity (SZN)
	Size-Zone Non-Uniformity Normalized (SZNN)
	Small Area Emphasis (SAE)
	Small Area High Gray Level Emphasis (SAHGLE)
	Small Area Low Gray Level Emphasis (SALGLE)
	Zone Entropy (ZE)
	Zone Percentage (ZP)
	Zone Variance (ZV)
**GLDM) (n=14)**	
	Dependence Entropy (DE)
	Dependence Non-Uniformity (DN)
	Dependence Non-Uniformity Normalized (DNN)
	Dependence Variance (DV)
	Gray Level Non-Uniformity (GLN)
	Gray Level Variance (GLV)
	High Gray Level Emphasis (HGLE)
	Large Dependence Emphasis (LDE)
	Large Dependence High Gray Level Emphasis (LDHGLE)
	Large Dependence Low Gray Level Emphasis (LDLGLE)
	Low Gray Level Emphasis (LGLE)
	Small Dependence Emphasis (SDE)

	Small Dependence High Gray Level Emphasis (SDHGLE)
	Small Dependence Low Gray Level Emphasis (SDLGLE)

GLCM: Gray Level Co-occurrence Matrix; GLRLM: Gray Level Co-occurrence Matrix; GLSZM: Gray Level Size Zone Matrix; GLDM: Gray Level Dependence Matrix.

The checklist from the radiomics reporting guidelines (https://arxiv.org/pdf/1612.07003.pdf) has been fully completed and included to facilitate a clear and comprehensive understanding of our methodology (Table 2).

Table 2 Checklist following the ISBI radiomics report guidelines

Topic	Item	Description	Details in this study
**Patient**			
Region of interest	1	Describe the region of interest that is being imaged.	Prostate gland
Patient preparation	2a	Describe specific instructions given to patients prior to image acquisition, e.g. fasting prior to imaging.	Not specific instructions given to patients.
	2b	Describe administration of drugs to the patient prior to image acquisition, e.g. muscle relaxants.	No administration of drugs.
	2c	Describe the use of specific equipment for patient comfort during scanning, e.g. ear plugs.	Ear plugs

Radioactive tracer	PET, SPECT	3a	Describe which radioactive tracer was administered to the patient, e.g. 18F-FDG.	NA
	PET, SPECT	3b	Describe the administration method.	NA
	PET, SPECT	3c	Describe the injected activity of the radioactive tracer at administration.	NA
	PET, SPECT	3d	Describe the uptake time prior to image acquisition.	NA
	PET, SPECT	3e	Describe how competing substance levels were controlled.	NA
Contrast agent		4a	Describe which contrast agent was administered to the patient.	NA
		4b	Describe the administration method.	NA

	4c	Describe the injected quantity of contrast agent.	NA
	4d	Describe the uptake time prior to image acquisition.	NA
	4e	Describe how competing substance levels were controlled.	NA
Comorbidities	5	Describe if the patients have comorbidities that affect imaging.	NA
**Acquisition**			
Acquisition protocol	6	Describe whether a standard imaging protocol was used, and where its description may be found.	Table 1 describes the imaging protocols.
Scanner type	7	Describe the scanner type(s) and vendor(s) used in the study.	Table 1 describes the scanner types.

Imaging modality		8	Clearly, state the imaging modality that was used in the study, e.g. CT, MRI.	MRI
Static/dynamic scans		9a	State if the scans were static or dynamic.	Static
	Dynamic scans	9b	Describe the acquisition time per time frame.	NA
	Dynamic scans	9c	Describe any temporal modelling technique that was used.	NA
Scanner calibration		10	Describe how and when the scanner was calibrated.	NA
Patient instructions		11	Describe specific instructions given to the patient during acquisition, e.g. breath holding.	During the scanning process, the patient is instructed to remain still and breathe steadily.

Anatomical motion correction		12	Describe the method used to minimise the effect of anatomical motion.	During the scanning process, the patient is instructed to remain still and breathe steadily.
Scan duration		13	Describe the duration of the complete scan or the time per bed position.	20-40 minutes
Tube voltage	CT	14	Describe the peak kilo voltage output of the X-ray source.	NA
Tube current	CT	15	Describe the tube current in mA.	NA
Time-of-flight	PET	16	State if scanner time-of-flight capabilities are used during acquisition.	NA
RF coil	MRI	17	Describe what kind RF coil used for acquisition, incl. vendor.	Body phased array coil

Scanning sequence	MRI	18a	Describe which scanning sequence was acquired.	T1WI, T2WI, DWI, and sometimes DCE
	MRI	18b	Describe which sequence variant was acquired.	No variant sequence was acquired.
	MRI	18c	Describe which scan options apply to the current sequence, e.g. flow compensation, cardiac gating.	No specific options were applied.
Repetition time	MRI	19	Describe the time in ms between subsequent pulse sequences.	Table 1 describes the TR values.
Echo time	MRI	20	Describe the echo time in ms.	Table 1 describes the TE values.
Echo train length	MRI	21	Describe the number of lines in k-space that are acquired per excitation pulse.	1 to 8

Inversion time	MRI	22	Describe the time in ms between the middle of the inverting RF pulse to the middle of the excitation pulse.	No inversion recovery sequence was used.
Flip angle	MRI	23	Describe the flip angle produced by the RF pulses.	90 degrees
Acquisition type	MRI	24	Describe the acquisition type of the MRI scan, e.g. 3D.	2D
k-space traversal	MRI	25	Describe the acquisition trajectory of the k-space.	Echo planar acquisition trajectory for DWI. Linear scan acquisition trajectory for T2WI.
Number of averages/ ex-citations	MRI	26	Describe the number of times each point in k-space is sampled.	1
Magnetic field strength	MRI	27	Describe the nominal strength of the MR magnetic field.	Table 1 describes the magnetic field strength.

**Reconstruction**				
In-plane resolution		28	Describe the distance between pixels, or alternatively the field of view and matrix size.	Table 1 describes the in-plane resolution.
Image slice thickness		29	Describe the slice thickness.	Table 1 describes the image slice thickness.
Image slice spacing		30	Describe the distance between image slices.	Table 1 describes the image slice spacing.
Convolution kernel	CT	31a	Describe the convolution kernel used to reconstruct the image.	NA
	CT	31b	Describe settings pertaining to iterative reconstruction algorithms.	NA
Exposure	CT	31c	Describe the exposure (in mAs) in slices containing the region of interest.	NA

Reconstruction method	PET	32a	Describe which reconstruction method was used, e.g. 3D OSEM.	NA
	PET	32b	Describe the number of iterations for iterative reconstruction.	NA
	PET	32c	Describe the number of subsets for iterative reconstruction.	NA
Point spread function modelling	PET	33	Describe if and how point-spread function modelling was performed.	NA
Image corrections	PET	34a	Describe if and how attenuation correction was performed.	NA

	PET	34b	Describe if and how other forms of correction were performed, e.g. scatter correction, randoms correction, dead time correction etc.	NA
Reconstruction method	MRI	35a	Describe the reconstruction method used to reconstruct the image from the k-space information.	Inverse Fourier transform was used to reconstruct the image from the k-space information.
	MRI	35b	Describe any artifact suppression methods used during reconstruction to suppress artifacts due to undersampling of k-space.	No specific artifact suppression methods used during reconstruction.
Diffusion-weighted imaging	DWI-MRI	36	Describe the b-values used for diffusion-weighting.	Table 1 describes the b values used for DWI.
**Image registration**				

Registration method		37	Describe the method used to register multi- modality imaging.	NA
**Image processing - data conversion**				
SUV normalisation	PET	38	Describe which standardised uptake value (SUV) normalisation method is used.	NA
ADC computation	DWI-MRI	39	Describe how apparent diffusion coefficient (ADC) values were calculated.	The ADC value for each voxel was calculated by applying the Stejskal-Tanner equation.
Other data conversions		40	Describe any other conversions that are performed to generate e.g. perfusion maps.	NA
**Image processing - post-acquisition processing**				

Anti-aliasing		41	Describe the method used to deal with anti-aliasing when down-sampling during interpolation.	NA
Noise suppression		42	Describe methods used to suppress image noise.	NA
Post-reconstruction smoothing filter	PET	43	Describe the width of the Gaussian filter (FWHM) to spatially smooth intensities.	NA
Skull stripping	MRI (brain)	44	Describe method used to perform skull stripping.	NA
Non-uniformity correction	MRI	45	Describe the method and settings used to perform non-uniformity correction.	We scaled image intensities to a standard range (e.g., 1-100) and aligned the median intensity across all patients to ensure uniformity in signal representation.

Intensity normalisation	46	Describe the method and settings used to normalise intensity distributions within a patient or patient cohort.	We scaled image intensities to a standard range (e.g., 1-100) and aligned the median intensity across all patients to ensure uniformity in signal representation.
Other post-acquisition processing methods	47	Describe any other methods that were used to process the image and are not mentioned separately in this list.	NA
**Segmentation**			
Segmentation method	48a	Describe how regions of interest were segmented, e.g. manually.	The ROI was segmented by a pre-trained AI model.
	48b	Describe the number of experts, their expertise and consensus strategies for manual delineation.	NA

	48c	Describe methods and settings used for semi-automatic and fully automatic segmentation.	The fully automatic segmentation model is described in reference 22.
	48d	Describe which image was used to define segmentation in case of multi-modality imaging.	NA
Conversion to mask	49	Describe the method used to convert polygonal or mesh-based segmentations to a voxel-based mask.	NA
**Image processing - image interpolation**			
Interpolation method	50a	Describe which interpolation algorithm was used to interpolate the image.	SitkBSpline algorithm was used to interpolate the image.

	50b	Describe how the position of the interpolation grid was defined, e.g. align by center.	Position of the interpolation grid was defined align by center.
	50c	Describe how the dimensions of the interpolation grid were defined, e.g. rounded to nearest integer.	Dimensions of the interpolation grid were defined rounded to nearest integer.
	50d	Describe how extrapolation beyond the original image was handled.	Padding, achieved by mirroring the edge values from the image, was used to extrapolate beyond the original image.
Voxel dimensions	51	Describe the size of the interpolated voxels.	The interpolated voxel size was 1 mm.
Intensity rounding CT	52	Describe how fractional Hounsfield Units are rounded to integer values after interpolation.	NA
**Image processing - ROI interpolation**			

Interpolation method	53	Describe which interpolation algorithm was used to interpolate the region of interest mask.	SitkBSpline algorithm was used to interpolate the ROI mask.
Partially masked voxels	54	Describe how partially masked voxels after interpolation are handled.	NA
**Image processing - re-segmentation**			
Re-segmentation methods	55	Describe which methods and settings are used to re-segment the ROI intensity mask.	NA
**Image processing - discretisation**			
Discretisation method	56a	Describe the method used to discretise image intensities.	Fixed bin width, e.g., each bin represents an intensity range of 5 units, was used to discretise image intensities.

	56b	Describe the number of bins (FBN) or the bin size (FBS) used for discretisation.	The number of bins used for discretisation was 20.
	56c	Describe the lowest intensity in the first bin for FBS discretisation.	The lowest intensity in the first bin for FBS was 1.
**Image processing - image transformation**			

Image filter	57	Describe the methods and settings used to filter images, e.g. Laplacian-of-Gaussian.	In the method part, we described three types of images were analyzed: "Original Images" representing unfiltered images, "LoG Images" obtained by applying the Laplacian of Gaussian filter, and "Wavelet Images" generated through a three-dimensional wavelet transformation using the PyWavelet package in the x, y, and z directions.
**Image biomarker computation**			

Biomarker set	58	Describe which set of image biomarkers is computed and refer to their definitions or provide these.	Age, total serum prostate specific antigen level (tPSA), clinical staging of the prostate cancer (cT), and ADC image features of the suspicious lesion in the prostate gland
IBSI compliance	59	State if the software used to extract the set of image biomarkers is able to reproduce the IBSI feature reference values.	We used PyRadiomics, a Python package dedicated to radiomic feature extraction from medical imaging, to extract the ADC image features. It is designed to be able to reproduce the IBSI feature reference values.
Robustness	60	Describe how robustness of the image biomarkers was assessed, e.g. test-retest analysis.	NA

Software availability	61	Describe which software and version was used to compute image biomarkers.	PyRadiomics v3.1.0 (https://github.com/AIM-Harvard/pyradiomics) was used to compute image biomarkers.
**Image biomarker computation - texture parameters**			

Texture matrix aggregation	62	Define how texture-matrix based biomarkers were computed from underlying texture matrices.	Texture-matrix based biomarkers were computed using various types of underlying texture matrices. The Gray Level Co-occurrence Matrix (GLCM) was used to assess pixel intensity correlations, the Gray Level Run Length Matrix (GLRLM) to analyze the length of consecutive runs of pixels with the same intensity, the Gray Level Size Zone Matrix (GLSZM) to evaluate the homogeneity of intensity areas, and the Gray Level Dependence Matrix (GLDM) to measure the dependence of pixel intensities

			on neighboring pixels. From each of these matrices, specific biomarkers were extracted to quantify different aspects of the image texture.
Distance weighting	63	Define how CM, RLM, NGTDM and NGLDM weight distances, e.g. no weighting.	In the computation of the Co-occurrence Matrix (CM) and Run Length Matrix (RLM), no weighting is applied to distances.
CM symmetry	64	Define whether symmetric or asymmetric cooccurrence matrices were computed.	Symmetric cooccurrence matrices were computed.

CM distance	65	Define the (Chebyshev) distance at which co-occurrence of intensities is determined, e.g. 1.	The Chebyshev distance used to determine the co-occurrence of intensities is set at 1.
SZM linkage distance	66	Define the distance and distance norm for which voxels with the same intensity are considered to belong to the same zone for the purpose of constructing an SZM, e.g. Chebyshev distance of 1.	In constructing a SZM, voxels with the same intensity are considered to belong to the same zone if they are within a Chebyshev distance of 1 from each other.

DZM linkage distance	67	Define the distance and distance norm for which voxels with the same intensity are considered to belong to the same zone for the purpose of constructing a DZM, e.g. Chebyshev distance of 1.	For constructing a DZM, voxels with the same intensity are considered to belong to the same zone if they are within a Chebyshev distance of 1 from each other.
DZM zone distance norm	68	Define the distance norm for determining the distance of zones to the border of the ROI, e.g. Manhattan distance.	Manhattan distance, which calculates the sum of the absolute differences of the Cartesian coordinates, was used to determine the distance of zones to the border of the ROI. This method measures distance based on a grid-like path, akin to navigating city blocks

NGTDM distance	69	Define the neighbourhood distance and distance norm for the NGTDM, e.g. Chebyshev distance of 1.	NA
NGLDM distance	70	Define the neighbourhood distance and distance norm for the NGLDM, e.g. Chebyshev distance of 1.	NA
NGLDM coarseness	71	Define the coarseness parameter for the NGLDM, e.g. 0.	NA
**Machine learning and radiomics analysis**			
Diagnostic and prognostic modelling	72	See the TRIPOD guidelines for reporting on diagnostic and prognostic modelling.	The performance of the model was reported in accordance with TRIPOD guidelines.

Comparison with known factors	73	Describe where performance of radiomics models is compared with known (clinical) factors.	In the result part, the DCA curve demonstrated superior performance of the deep-radiomics model compared to the conventional radiomics model and the clinical model across all risk thresholds.
Multicollinearity	74	Describe where the multicollinearity between image biomarkers in the signature is assessed.	In the methods part, the Pearson correlation coefficient was used to deal with the multicollinearity.
Model availability	75	Describe where radiomics models with the necessary pre-processing information may be found.	In the methods part and the supplementary materials, we provided the pre-processing information.
Data availability	76	Describe where imaging data and relevant meta-data used in the study may be found.	The authors are not allowed to share the data publicly.

Part 2 Feature Selection

After feature extraction, several additional steps were performed for feature selection.

First, Z score normalization was applied to rescale the extracted features. This method utilizes the mean (μ) and standard deviation (σ) of the original data to standardize the data. The resulting data conform to a standard normal distribution, meaning that the mean is 0 and the standard deviation is 1. The transformation is applied using the formula $x^* = \frac{x-\mu}{\delta}$ (where μ is the mean of all sample data and σ is the standard deviation of all sample data).

Subsequently, Pearson correlation coefficients (PCCs) were calculated to identify highly correlated features. The PCC is used to measure the degree of linear correlation between two features. If the PCC value between two features exceeded 0.99, one of the features was removed at random. This step reduces the dimensionality of the feature set while retaining its original classification power.

Then, Analysis of Variance (ANOVA) was employed to select features for the radiomics model. The F-value for each feature was calculated to evaluate its relationship with the label. Features were ranked based on their corresponding F-values, and the top 20 features were selected for model building.

During the model building process, we tested and selected different numbers of features from the top 20 features to train the models. For instance, we started by selecting 1 feature and then 2 features, followed by 3 features, and so on, for up to 20 features. A corresponding model was trained for each different number of selected features. Among these models, we chose the one with the highest average AUC value during cross-validation as the final model. In this study, the final radiomics model included 4 features, while the final deep-radiomics model included 3 features.

Part 3 Internal Validation Methods

In our study, we employed an internal validation framework using 5-fold cross-validation to ensure the reliability and generalizability of our predictive models, both radiomic and deep-radiomics. This process involved several key steps:

1. Data partitioning: Initially, the training dataset was subdivided into five distinct subsets.

2. Sequential Model Training and Validation: For each fold in the 5-fold cross-validation, four subsets were combined to form the training data, and the remaining subset served as the validation data. This process was repeated five times, with each subset serving as the validation data exactly once. This approach ensures that every data point in the training set is used for both training and validation, thereby enhancing the model's robustness.

3. Performance Metrics Calculation: During each fold of the cross-validation, key performance metrics (AUC) were computed.

4. Model Selection and Optimization: After completing the 5-fold cross-validation, we aggregated the performance metrics across all folds to obtain an overall performance profile for each model. The model exhibiting the highest AUC was selected as the optimal model.

5. Final Evaluation on the Test Set: The chosen model was then subjected to a final evaluation on the independent test set.

Part 4 Model building

The eXtreme gradient boosting (XGBoost) algorithm was employed as the classifier in this study (sklearn 0.24.1, Xgboost 1.4.1), with log loss as the loss function. The settings of the hyperparameters were as follows:

max_depth=None,

learning_rate=None,

n_estimators=100,

verbosity=None,

objective=None,

booster=None,

tree_method=None,

n_jobs=None,

gamma=None,

min_child_weight=None,

max_delta_step=None,

base_score=None,

random_state=None,

missing=np.nan,

num_parallel_tree=None,

monotone_constraints=None,

interaction_constraints=None,

importance_type="gain",

gpu_id=None,

validate_parameters=None.
